# Plasma Haptoglobin Concentrations Are Elevated in Patients with Coronary Artery Disease

**DOI:** 10.1371/journal.pone.0076817

**Published:** 2013-10-09

**Authors:** Chin-Wei Lee, Tsai-Mu Cheng, Chih-Pei Lin, Ju-Pin Pan

**Affiliations:** 1 Division of Cardiology, Department of Medicine, Taipei Veterans General Hospital, Tao-Yuan Branch, Tao-Yuan, Taiwan, ROC; 2 Graduate Institute of Translational Medicine, College of Medicine and Technology, Taipei Medical University, Taipei, Taiwan, ROC; 3 Biochemistry Section, Department of Pathology and Laboratory Medicine, Taipei Veterans General Hospital, Taipei, Taiwan, ROC; 4 Institute of Biotechnology in Medicine, National Yang-Ming University, Taipei, Taiwan, ROC; 5 Division of Cardiology, Department of Medicine, Taipei Veterans General Hospital, Taipei, Taiwan, ROC; 6 Faculty of Medicine, School of Medicine, National Yang-Ming University, Taipei, Taiwan, ROC; Kaohsiung Chang Gung Memorial Hospital, Taiwan

## Abstract

Inflammation underlies the development and progression of coronary artery plaques. Haptoglobin (Hp) is an acute phase protein, the synthesis of which is increased during inflammation. The aim of this study was to investigate plasma Hp concentrations and phenotype in patients with coronary artery disease (CAD). We recruited 359 patients with fixed luminal stenosis ≥50% in at least one coronary artery (CAD group) and 83 patients with luminal stenosis ≤40%, normal ejection fraction, and normal regional wall motion (control group). Plasma Hp concentrations were measured using a phenotype-specific enzyme-linked immunosorbent assay. Hp phenotype was determined by native polyacrylamide gel electrophoresis. Plasma lipid concentrations were measured. Plasma Hp concentrations were significantly higher in the CAD compared with the control group (262.4±144.2 vs 176.0±86.7 ng/mL, *P*<0.001); however, there was no between group difference in the distribution of Hp phenotype (1-1 = 7.5% vs 7.2%; 2-1 = 40.4% vs 42.2%; 2-2 = 52.1% vs 50.6%). Stepwise multivariate logistic regression revealed that high Hp concentrations (odds ratio [OR] = 5.865), male sex (OR = 3.689), hypertension (OR = 2.632), diabetes mellitus (OR = 3.300), and low-density lipoprotein concentrations (OR = 1.480) were independently associated with CAD (all *P*<0.05). Hp phenotype was not associated with CAD. Plasma Hp concentrations were significantly correlated with the severity of luminal stenosis (r = 0.236, *P*<0.001). Our findings suggest that plasma Hp concentrations may be elevated in patients with CAD. There does not appear to be any relationship between Hp phenotype and CAD.

## Introduction

Inflammation appears to play a key role in mediating coronary artery disease (CAD), a leading cause of death in the developed world [1]. Of note, a large body of evidence has demonstrated that inflammation plays a pivotal role in atherogenesis, unstable progression, and eventual disruption of atheroma plaques [2-4]. The mediators of this inflammation continue to be investigated in an effort to better understand the etiology, diagnosis, and treatment of CAD.

Haptoglobin (Hp) is the major hemoglobin (Hb) binding protein, as well as being an acute phase protein, the expression of which is increased during inflammation [5]. The primary function of Hp is the binding of extracorpuscular free Hb, which attenuates the oxidative and inflammatory effects of Hb [6]. The Hb-Hp complex is rapidly removed by the CD163 scavenger receptor on monocytes and macrophages [7]. Hp also exerts antioxidant effects by directly inhibiting the oxidation of low-density lipoprotein (LDL) [8]. There are two Hp alleles, Hp 1 and 2. In humans, homozygosity for the Hp 2 allele is designated 2-2, homozygosity for the Hp 1 allele is designated 1-1, and heterozygosity for the Hp allele is designated 2-1 [6,9]. 

Current evidence concerning the relationship between Hp phenotypes and cardiovascular disease is inconclusive. Findings from an in vitro study suggest that Hp 1-1 protein is superior to Hp 2-2 protein in attenuating the oxidative action of Hb [10]. Further, the findings from an in vivo study revealed that atherosclerotic plaques from Hp 2-2 mice exhibited increased lipid peroxidation and macrophage accumulation, and contained more iron compared with plaques from Hp 1-1 mice. This finding suggests that the Hp genotype may play a critical role in the oxidative and inflammatory response to intraplaque hemorrhage [11]. A number of reports concerning human studies have also indicated that the Hp 2-2 phenotype may be associated with an increased risk of myocardial infarction, stroke, and cardiovascular death [9,12-15]. In contrast, findings from the Framingham offspring cohort suggest that there is no relationship between Hp phenotype and the prevalence of coronary heart disease [16]. 

Although a number of studies have examined the Hp phenotype in the context of cardiovascular disease, no study date has examined the relationship between human plasma Hp concentrations and CAD. This may be because the purification process involved in the Hp immunoassay is challenging [17,18]. Of note, we have previously reported that plasma Hp concentrations are elevated in patients with abdominal aortic aneurysm (perhaps due to inflammation in the destructive elastic laminae of arteries) [19]. The aim of this study was to investigate plasma Hp concentrations and Hp phenotype in a cohort of Chinese patients with coronary artery disease (CAD).

## Methods

### Study Participants

The study participants were genetically unrelated patients of Chinese ancestry who were treated consecutively between January 2003 and December 2005 in the Division of Cardiology at Taipei Veterans General Hospital. All participants received coronary angiography (CAG) under the suspicion of coronary artery disease (CAD) with a positive thallium myocardial perfusion scan. All angiograms were independently evaluated by two medical imaging specialists who were unaware of the participants’ medical histories. Multiple views from different angles were examined, and the degree of CAD was ascribed according to the coronary arterial segment with the most severe stenosis per the American Heart Association recommendations [20]. CAD was defined as a fixed stenotic lesion with luminal narrowing ≥ 50% in at least one of the major or minor coronary arteries. Patients who had luminal stenosis ≤ 40% (as determined by CAG), normal ejection fraction, and normal regional wall motion on left ventriculography were assigned to the control group. The left anterior descending artery, left circumflex artery, and right coronary artery were examined to evaluate the number of stenotic coronary arteries (none to three). All participants were free of unstable angina pectoris. Patients who had a documented myocardial infarction event within three months before undergoing coronary angiography were excluded. The study was approved by the Institutional Review Board of Taipei Veterans General Hospital. All patients who were enrolled in the study provided written informed consent.

### Clinical Chemistry

Overnight fasting blood samples were obtained from all patients before catheterization and mixed with 0.1% ethylenediamine tetraacetic acid for measurement of total cholesterol, high-density lipoprotein (HDL), LDL, and triglyceride concentrations using standard techniques. Plasma C-reactive protein (CRP) concentrations were determined by IMMAGE high sensitivity CRP assay (Beckman Coulter, Inc, Fullerton, CA). 

### Hp Phenotyping

Hp phenotyping was carried out as previously described [21] using native polyacrylamide gel electrophoresis (PAGE) with hemoglobin-supplemented plasma. Briefly, overnight fasting blood samples were obtained from all patients before catheterization and mixed with 0.1% ethylenediamine tetraacetic acid. Plasma was isolated immediately by centrifuge then stored at -20°C. 7 μL of plasma was premixed with 5 μL of 8 mg/mL hemoglobin and equilibrated with 3 μL of sample buffer (0.625 mol/L Tris-base, pH 6.8, 50% glycerol (v/v), and 0.125 mg/L bromophenol blue). The mixture was run on a 7% native polyacrylamide gel (pH 8.8), with 5.5% polyacrylamide (26.5:1; acrylamide: bisacrylamide) used as a top stacking gel (pH 6.8). Electrophoresis was performed at an initial voltage of 120 V, which was increased up to 150 V when the dye front reached the separating gel. After electrophoresis, the Hp‑hemoglobin complex was visualized by shaking the gel in freshly prepared peroxidase substrate (0.05% 3,3′-diaminobenzidine [w/v] and 0.07% hydrogen peroxide [v/v] in phosphate buffered saline). The results were confirmed by Western blotting using an α-chain specific monoclonal antibody.

### Hp Purification

Hp was purified from plasma by chromatography on a mAb-based affinity column followed by high-performance liquate chromatography (HPLC) as described previously [18]. Briefly, 2 mL of filtered human plasma was loaded onto the antibody affinity-column (10 mL bed volume) at room temperature. The column was then washed with 50 mL of 20 mmol/L phosphate buffer containing 0.2 mol/L NaCl (pH 7.4), and then eluted with 50 mL of freshly prepared 0.15 mol/L NaCl solution (pH 11). Fractions (5 mL) were collected in tubes containing 0.25 mL of 1 mol/L Tris–HCl buffer (pH 6.8) to immediately neutralize the base. Pooled fractions containing Hp were then concentrated to a final volume of 1 mL using Centricon tubes (Millipore, Cork, Ireland) and filtered through a 0.45 μm membrane. Gel-filtration HPLC with a Superose-12 column (1×30 cm) (Pharmacia, Uppsala, Sweden) was then performed. The homogeneity of each isolated Hp type was greater than 95% as judged by sodium dodecyl sulfate-PAGE.

### Measurement of Human Free Hp Plasma Concentrations

Human free Hp plasma concentrations were measured using a phenotype-matched standard sandwich enzyme-linked immunosorbent assay as described in our previous publication [18]. 

### Statistical Analysis

Patients’ demographic and clinical characteristics are summarized as mean ± standard deviation for continuous data and number (percent) for categorical data. Continuous data were compared between groups by two-sample t-test or Mann-Whitney U, whereas categorical data were compared between groups by Pearson Chi-square test or Fisher’s exact test. The optimal Hp concentration cut-off point for predicting CAD was determined based on maximization of the Youden index (Sensitivity+Specificity-1) using receiver operating characteristic (ROC) curve analysis. Pearson correlation analysis was performed to identify the correlation between Hp concentration and stenotic percentage and between Hp and CRP concentrations. Simple logistic regression analysis was performed to identify associations between participants’ characteristics and CAD. Variables with significance (*P* < 0.05) in the simple logistic regression analysis were entered into multiple logistic regression analysis. Results are presented as estimated odds ratios (OR) with respective 95% confidence intervals (95% CI) and *P* values. All statistical assessments were two-tailed and considered significant if *P* < 0.05. Statistical analyses were performed using SPSS 15.0 statistics software (SPSS Inc, Chicago, IL).

## Results

A total of 442 participants, 359 in the CAD group and 83 in control group, were included in the study. Table 1 summarizes the baseline characteristics of these participants. Several baseline characteristics demonstrated significant between group differences. A significantly higher proportion of participants in the CAD group were men, had hypertension, had diabetes mellitus, were smokers, or took angiotensin converting enzyme inhibitors compared with participants in control group (all *P* < 0.05). HDL concentrations were significantly lower and LDL and Hp concentrations were significantly higher in the CAD group compared with the control group (all *P* < 0.05). 

**Table 1:** Baseline characteristics of study participants (N = 442).

		CAD	Control	
Variables		(n = 359)	(n = 83)	*P* Value
Age (years)		68.6 ± 8.6	69.0 ± 7.8	0.756
Male sex		325 (90.5)	61 (73.5)	<0.001^*^
BMI (kg/m^2^)		25.36 ± 3.50	25.99 ± 3.34	0.168
Hypertension		232 (64.6)	31 (37.8)	<0.001^*^
Diabetes Mellitus		96 (26.7)	7 (8.5)	<0.001^*^
Smoking		184 (51.4)	25 (30.9)	0.001^*^
Drinking		114 (31.8)	19 (23.5)	0.142
Hemoglobin (g/dL)		13.0 ± 1.8	13.1 ± 1.7	0.737
CRP (mg/L)		0.88 ± 1.67	0.62 ± 1.60	<0.001^*^
Lipid profile (mg/dL)	TC	192.9 ± 45.8	189.1 ± 38.7	0.477
	TG	161.9 ± 112.0	157.3 ± 154.9	0.060
	HDL	37.5 ± 11.7	40.6 ± 12.3	0.030^*^
	LDL	124.5 ± 44.2	112.4 ± 34.9	0.020^*^
Prior MI^a^	Yes	144 (40.1)	ND	NA
	No	215 (59.9)	ND	
Number of diseased vessels	One	105 (29.2)	ND	NA
	Two	98 (27.3)	ND	
	Three	156 (43.5)	ND	
Medications	ARB	101 (28.1)	15 (18.1)	0.062
	ACEI	142 (39.6)	20 (24.1)	0.008^*^
	Statin	94 (26.2)	16 (19.3)	0.190
	Beta-blocker	60 (16.7)	11 (13.3)	0.439
Hp concentration (ng/mL)		262.4 ± 144.2	176.0 ± 86.7	<0.001^*^
Hp phenotype	1/1	27 (7.5)	6 (7.2)	0.957
	2-1	145 (40.4)	35 (42.2)	
	2/2	187 (52.1)	42 (50.6)	

Data are presented as mean ± standard deviation or number (percent).

ACEI: angiotensin converting enzyme inhibitor; ARB: angiotensin receptor blocker; BMI: body mass index; CAD: coronary artery disease; CRP, C-reactive protein; HDL: high-density lipoprotein; Hp: haptoglobin; LDL: low-density lipoprotein; MI: myocardial infarction; NA: not applicable; ND: not determined; TC: total cholesterol; TG: triglyceride.

^a^ greater than or equal to three months before the start of the study.

^*^ Indicates a statistically significant between group difference (*P* < 0.05).

**Figure 1:**
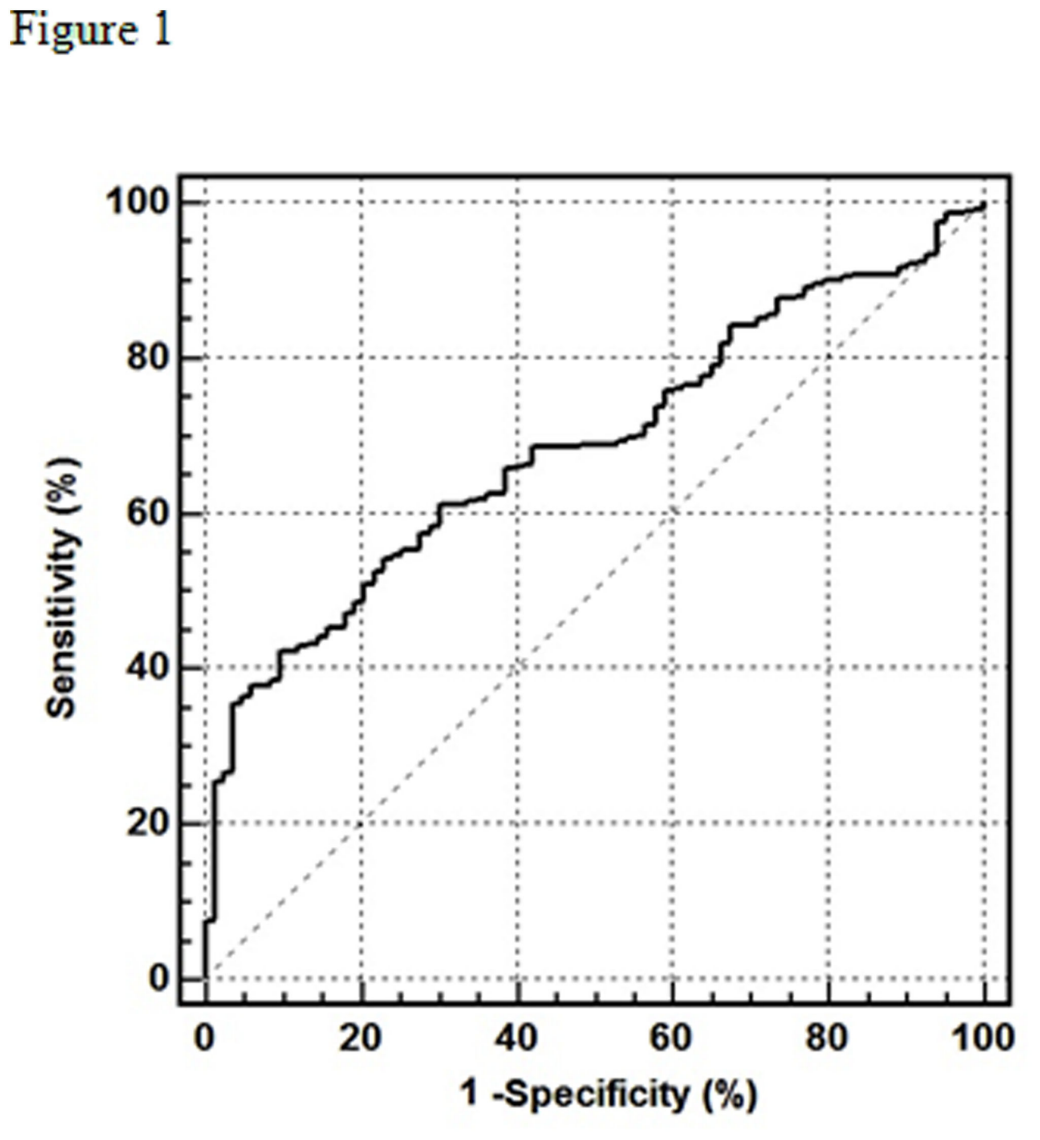
Receiver operating characteristic (ROC) curve for the optimal haptoglobin concentration cut-off for identifying the presence / absence of coronary artery disease.

The Hp concentration cut-off for CAD, determined using ROC curve analysis (Figure 1), was found to be 288.4 ng/mL (specificity = 42.1%; specificity = 90.4%, positive predictive value = 95.0%; negative predictive value = 90.4%). On the basis of this findings, participants were categorized for further analysis as having low (≤ 288.4 ng/mL) or high (> 288.4 ng/mL) Hp concentrations.

Table 2 summarizes the participants’ baseline characteristics by group and Hp concentration (low or high). Very few participants in the control group had high Hp concentrations. In both groups, participants with low Hp concentrations had significantly higher hemoglobin concentrations (both *P* < 0.05). In the CAD group, there was a significant difference in the proportion of participants with different Hp phenotypes (*P* < 0.001). Of note, a much higher proportion of participants with low Hp concentrations had the 1-1 Hp phenotype compared with participants with high Hp concentrations. 

**Table 2:** Comparison of participants’ characteristics by coronary artery disease status and haptoglobin concentration (low or high).

		CAD				Control		
Variable		Low, Hp ≤288.4 ng/mL (n = 208)	High, Hp >288.4 ng/mL (n = 151)	*P*		Low, Hp ≤288.4 ng/mL (n = 75)	High, Hp >288.4 ng/mL (n = 8)	*P*
Age (years)		68.52 ± 8.00	68.8 ± 9.5	0.777		69.36 ± 7.82	65.13 ± 7.22	0.147
Male sex		190 (52.9)	135 (41.5)	0.535		53 (86.9)	8 (13.1)	0.102
BMI (kg/m^2^)		25.34 ± 3.40	25.40 ± 3.64	0.865		26.04 ± 3.29	25.50 ± 4.07	0.745
Hypertension		129 (55.6)	103 (44.4)	0.249		30 (96.8)	1 (3.2)	0.248
Diabetes Mellitus		55 (57.3)	41 (42.7)	0.881		6 (85.7)	1 (14.3)	0.527
Smoking		101 (54.9)	83 (45.1)	0.248		24 (96.0)	1 (4.0)	0.424
Drinking		64 (56.1)	50 (43.9)	0.638		18 (94.7)	1 (5.3)	0.673
Hemoglobin (g/dL)		13.24 ± 1.59	12.7 ± 2.1	0.013^*^		13.26 ± 1.55	11.57 ± 2.08	0.010^*^
CRP (mg/L)		0.63 ± 1.37	1.22 ± 1.97	<0.001*		0.65 ± 1.69	0.35 ± 0.26	0.365
Lipid profile (mg/dL)	TC	194.83 ± 45.34	190.3 ± 46.5	0.355		189.24 ± 37.72	187.38 ± 50.01	0.898
	TG	169.54 ± 127.62	151.3 ± 85.0	0.129		163.00 ± 160.57	103.63 ± 69.77	0.072
	HDL	37.79 ± 12.99	37.1 ± 9.7	0.572		40.47 ± 12.82	42.13 ± 6.69	0.562
	LDL	126.73 ± 44.28	121.5 ± 44.0	0.273		112.77 ± 34.35	109.13 ± 41.87	0.752
Prior MI		79 (54.9)	65 (45.1)	0.334		ND	ND	NA
Number of diseased vessels	One	63 (60.0)	42 (40.0)	0.846		ND	ND	NA
	Two	57 (58.2)	41 (41.8)			ND	ND	
	Three	88 (56.4)	68 (43.6)			ND	ND	
Stenotic percentage^b^		90.80 ± 12.13	90.25 ± 12.34	0.609		5.67 ± 11.69	ND^c^	NA
Hp concentration (ng/mL)		165.25 ± 72.85	396.3 ± 105.6	<0.001^*^		159.37 ± 71.62	332.23 ± 53.67	<0.001^*^
Hp phenotype	1-1	25 (92.6)	2 (7.4)	<0.001^*^		6 (100)	0 (0)	1.000
	2-1	55 (37.9)	90 (62.1)			31 (88.6)	4 (11.4)	
	2-2	128 (68.4)	59 (31.6)			38 (90.5)	4 (9.5)	
Medications	ARB	54 (53.5)	47 (46.5)	0.283		13 (86.7)	2 (13.3)	0.631
	ACEI	82 (57.7)	60 (42.3)	0.952		18 (90.0)	2 (10.0)	1.000
	Statin	51 (54.3)	43 (45.7)	0.400		15 (93.8)	1 (6.3)	1.000
	Beta-blocker	32 (53.3)	28 (46.7)	0.428		10 (90.9)	1 (9.1)	1.000

Data are presented as mean ± standard deviation or number (percent).

ACEI: angiotensin converting enzyme inhibitor; ARB: angiotensin receptor blocker; BMI: body mass index; CAD: coronary artery disease; CRP: C-reactive protein; HDL: high-density lipoprotein; Hp: haptoglobin; LDL: low-density lipoprotein; MI: myocardial infarction; NA: not applicable; ND: not determined; TC: total cholesterol; TG: triglyceride.

a greater than or equal to three months before the start of the study

b The stenotic percentage was the percent stenosis of the most affected artery.

c The stenotic percentage was equivalent to zero for all the patients in the control group with a high Hp concentration.

* Indicates a significant within group difference in Hp concentrations (*P* < 0.05).

Overall, there was a significant positive correlation between Hp concentration and stenotic percentage (r = 0.236, *P* < 0.001). However, there were no significant correlations between these variables for either the CAD or control group alone (data not shown).

There was a significant correlation between Hp and CRP concentrations in the CAD group (r = 0.386, *P* < 0.001), but not in the control group (r = 0.197, *P* = 0.112).

Simple logistic regression analysis revealed that CAD was significantly associated with high Hp concentrations, male sex, hypertension, diabetes mellitus, smoking, low HDL concentrations, and high LDL concentrations (all *P* < 0.05, Table 3). Subsequent multiple logistic regression analysis revealed that CAD was significantly associated with high Hp concentrations, male sex, hypertension, diabetes mellitus, and high LDL concentrations (all *P* < 0.05, Table 3). The C-statistic for the multiple logistic regression model was 0.805 (95% CI: 0.757 to 0.853, *P* < 0.001); Meanwhile, the C-statistic for the simple logistic regression model was ranged from 0.582 (95%CI: 0.508 to 0.652, *P*=0.023) to 0.662 (95CI: 0.604 to 0.720, *P* < 0.001) for those variables selected into multiple logistic regression analysis. For the simple logistic regression model, the -2ln likelihood ratio was 396.3 with the inclusion of Hp concentration as a variable; meanwhile, the -2ln likelihood ratio was ranged from 403.3 to 419.6 for those variables selected into multiple logistic regression model. For the multiple logistic regression model, the -2ln likelihood ratio was 354.2 without the inclusion of Hp concentration as a variable and 327.8 with the inclusion of Hp concentration as a variable.

**Table 3:** Factors associated with coronary artery disease: Simple and multiple logistic regression analyses.

Variable		Simple Logistic Regression			Multiple Logistic Regression	
		OR (95% CI)	*P* value		OR (95% CI)	*P* value
Hp concentration^a^	Low	Reference			Reference	
	High	6.806 (3.188, 14.531)	<0.001^*^		5.865 (2.672, 12.874)	<0.001^*^
Age (years)		0.962 (0.756, 1.225)	0.755		-	
Male sex	Female	Reference			Reference	
	Male	3.447 (1.888, 6.294)	<0.001^*^		3.689 (1.683, 8.087)	0.001^*^
BMI (kg/m^2^)		0.840 (0.666, 1.059)	0.141		-	
Hypertension	No	Reference			Reference	
	Yes	3.029 (1.844, 4.976)	<0.001^*^		2.632 (1.518, 4.564)	0.001^*^
Diabetes mellitus	No	Reference			Reference	
	Yes	3.911 (1.741, 8.783)	0.001^*^		3.300 (1.357, 8.024)	0.008^*^
Smoking	No	Reference			Reference	
	Yes	2.369 (1.415, 3.964)	0.001^*^		1.654 (0.911, 3.002)	0.098
Drinking	No	Reference				
	Yes	1.518 (0.867, 2.658)	0.144		-	
Hemoglobin (g/dL)		0.957 (0.740, 1.237)	0.736		-	
CRP (mg/L)		1.247 (0.854, 1.821)	0.254		-	
Lipid profile (mg/dL)	TC	1.094 (0.855, 1.400)	0.476		-	
	TG	1.041 (0.808, 1.341)	0.754		-	
	HDL	0.785 (0.625, 0.984)	0.036^*^		0.966 (0.753, 1.240)	0.786
	LDL	1.371 (1.049, 1.792)	0.021^*^		1.480 (1.096, 1.997)	0.010^*^
Hp phenotype	1-1	Reference				
	2-1	0.921 (0.353, 2.401)	0.866		-	
	2-2	0.989 (0.384, 2.548)	0.982		-	

The ORs for continuous variables are per standard deviation increase.

BMI: body mass index; CI: confidence interval; CRP: C-reactive protein; HDL: high-density lipoprotein; Hp: haptoglobin; LDL: low-density lipoprotein; OR: odds ratio; TC: total cholesterol; TG: triglyceride.

High Hp concentration, Hp > 288.4 ng/mL; Low Hp concentration, Hp ≤ 288.4 ng/mL.

a High Hp concentration, Hp > 288.4 ng/mL; Low Hp concentration, Hp ≤ 288.4 ng/mL.

* Indicates a significant association (*P* < 0.05).

## Discussion

To our knowledge, this is the first study to examine plasma Hp concentrations in patients with CAD. Of note, we found that plasma Hp concentrations were significantly higher in participants with CAD compared with participants who did not have CAD, and that high plasma Hp concentrations were independently associated with CAD. We did not find any between group differences in the distribution of Hp phenotypes. 

As already noted, plasma Hp concentrations are known to be increased during inflammation, and indeed by a number of other physiological processes / states, including hemolysis, ineffective erythropoiesis, liver disease, and late pregnancy [22]. Our finding that plasma Hp concentrations were significantly increased in participants with CAD is consistent with a previous report that plasma concentrations of the soluble form of CD163 are elevated in patients with CAD [23]. The current finding is also in keeping with our previously reported finding that plasma Hp concentrations are increased in patients with abdominal aortic aneurysm [19]. We suggest that the increased plasma Hp concentrations in CAD may be a regulatory response to atherosclerotic progression. Hp may also play a proatherogenic role, as suggested by the recently reported finding that Hp acts as a chemoattractant to pre-B lymphocytes and monocytes in inflammatory adipose tissue [24]. 

Of note, we found that plasma Hp concentrations, along with traditional risk factors such as hypertension, diabetes, smoking, and LDL concentration, were a significant predictor of CAD. A number of investigators have previously attempted to identify inflammatory biomarkers of CAD. Indeed, in a systematic review, Hamirani and colleagues reported that concentrations of a number of inflammatory mediators, including CRP, fibrinogen, metallic metalloproteinase-9, monocyte chemotactic protin-1, resistin, lipoprotein-associated phospholipase A2, interleukin-6, tumor necrosis factor-alpha, and beta-fibroblast growth factor, were positively correlated with atherosclerosis [25]. Unfortunately, these relationships disappeared after correction for traditional risk factors of CAD [25]. In contrast, we found that plasma Hp concentration remained a significant independent predictor of CAD after controlling for other variables in multivariate analysis. Hence, assessing plasma Hp concentrations, along with other factors, may prove to be of use in the future for the diagnosis / monitoring of CAD.

Interestingly, we did not find any significant relationship between Hp phenotype and CAD. This could be considered somewhat surprising given the evidence from other studies that the Hp 2-2 phenotype may be associated with cardiovascular disease. Indeed, individuals with the Hp 2-2 phenotype appear to have reduced clearance of the macrophage-Hp-Hb complex, which affects iron deposition, oxidative stress, and active macrophage accumulation [26]. These changes would be consistent with an increased risk of atherosclerotic cardiovascular disease. Other studies have reported that the Hp 2-2 phenotype is associated with an increased risk of peripheral arterial occlusive disease [27], diabetic nephropathy [28], acute myocardial infarction, stroke, and heart failure [12]. Indeed, we have also found that the Hp 2-2 phenotype is more common among patients with abdominal aortic aneurysm compared with patients who do not have this condition [19]. However, the findings from a number of other studies do not support an association between Hp phenotype and cardiovascular disease [16,29,30]. Indeed, Carter et al. [22] concluded that even though biological and functional differences may exist between Hp phenotypes, the clinical associations with these differences may be marginal. Of note, we found that a significantly higher proportion of participants with low Hp concentrations had the Hp 1-1 phenotype compared with those who had high Hp concentrations. This finding is consistent with Hp 1-1 having the strongest affinity for binding Hp [10]. Given the purported link between Hp and CAD, one may have surmised that patients with the Hp 1-1 phenotype would have a decreased risk of CAD because of their lower plasma Hp concentrations. However, no such decreased risk was identified in our regression analysis, presumably because a large proportion of participants with low Hp concentrations had the Hp 2-2 phenotype. Taken together, our findings do not support the existence of an association between Hp phenotype and CAD. 

We found that plasma Hp concentrations were significantly associated with the extent of stenosis for the overall study population, but not the CAD or control group alone. However, the strength of correlation was not particularly high (r = 0.236). This weak positive correlation, and indeed the lack of any correlation between Hp concentration and the extent of stenosis among participants with CAD, is perhaps not unsurprising given that Hp is a global marker of inflammation and is not specific for CAD. 

Our study has several limitations that warrant acknowledgment. Of note, the control group of participants was recruited from a hospital-based population and was relatively small in size. Further, all participants in the control group had positive thallium myocardial perfusion scans. Furthermore, several differences in baseline characteristics also existed between study participants in the two groups. These cohort characteristics and differences may have introduced bias and affected the results of our study to some extent. Hence, a more appropriately controlled study is warranted to confirm the findings reported herein. 

In summary, we have reported that plasma Hp concentrations are significantly increased in patients with CAD compared with patients who do not have CAD, and that plasma Hp concentrations are independently associated with CAD. We did not detect any association between Hp phenotype and CAD. Further studies are needed to better elucidate the apparent link between Hp and CAD, and to examine the potential diagnostic / monitoring significance of such a link. Refinement / simplification of the methodology for assessing plasma Hp concentration is also essential for wider clinical application.
