# Correction: Plasma Haptoglobin Concentrations Are Elevated in Patients with Coronary Artery Disease

**DOI:** 10.1371/annotation/5ae7dfbe-64c8-40d1-aff2-42ee2fc9408c

**Published:** 2014-01-06

**Authors:** Ching-Wei Lee, Tsai-Mu Cheng, Chih-Pei Lin, Ju-Pin Pan

The name of the first author was spelled incorrectly. The correct name is: Ching-Wei Lee. 

